# Supplementary material for: Empirical methods for controlling false positives and estimating confidence in ChIP-Seq peaks
Source: BMC Bioinformatics. 2008 Dec 5;9:523. doi: 10.1186/1471-2105-9-523 (PMC2628906; doi:10.1186/1471-2105-9-523)
Supplement: Additional File 1 — A variety of html documents from the USeq web site detailing the available applications, their best usage, output file type descriptions, command line menus, etc. [file 1471-2105-9-523-S1.zip › USeqUserGuides/JavaDoc.zip!/JavaDoc/constant-values.html]

Constant Field Values


|  |  |  |
| --- | --- | --- |
| **Overview** \| Package \| Class \| Use \| **Tree** \| **Deprecated** \| **Index** \| **Help** | |  |
| PREV   NEXT | **FRAMES**    **NO FRAMES**     **All Classes** |  |


---


# Constant Field Values


---

**Contents**

- bioroot.\*- cgar.\*- edu.utah.\*- trans.bkgrnd.\*- trans.tpmap.\*- util.amazon.\*- util.apps.\*- util.bio.\*

bioroot.\*

| bioroot.Util | | |
| --- | --- | --- |
| `public static final java.lang.String` | `contactEmail` | `"info@bioroot.org"` |
| `public static final int` | `demoUserId` | `2` |
| `public static final java.lang.String` | `filepath` | `"/home/orgbioro/public_html/"` |
| `public static final java.lang.String` | `fullPathToBlast` | `"/home/orgbioro/public_html/Blast/"` |
| `public static final java.lang.String` | `HTMLPath` | `"http://bioroot.org/"` |
| `public static final java.lang.String` | `IPPort` | `"bioroot.org"` |
| `public static final int` | `maxFileSize` | `250000` |
| `public static final java.lang.String` | `SMTPAccount` | `"mail.bioroot.org"` |
| `public static final java.lang.String` | `smtpHostName` | `"mail.bioroot.org"` |
| `public static final java.lang.String` | `smtpPassword` | `"TGXWJ42R"` |
| `public static final java.lang.String` | `smtpUser` | `"orgbioro"` |

bioroot.antibody.\*

| bioroot.antibody.AntibodyBase | | |
| --- | --- | --- |
| `public static final java.lang.String` | `antibodyPrepFeatureNames` | `"Ab Prep Name,Date,Storage Location,Purification,User Comments,Recipients,Last User,Remaining Aliquots,Ab Prep File,Ab Prep Notes,Host Species,"` |
| `public static final java.lang.String` | `useDilutionFeatureNames` | `"Use,Dilution,Use Dil Notes,"` |

cgar.\*

| cgar.Util | | |
| --- | --- | --- |
| `public static final java.lang.String` | `baseURL` | `"http://localhost:8080/CGAR/"` |
| `public static final java.lang.String` | `contactEmail` | `"bioinformaticscore@utah.edu"` |
| `public static final java.lang.String` | `databaseDriver` | `"com.mysql.jdbc.Driver"` |
| `public static final java.lang.String` | `databaseName` | `"cgar"` |
| `public static final java.lang.String` | `databasePassword` | `"snoNowPlzz"` |
| `public static final java.lang.String` | `databaseURL` | `"jdbc:mysql://localhost:3306/"` |
| `public static final java.lang.String` | `databaseUser` | `"cgarAdmin"` |

edu.utah.\*

| edu.utah.seq.analysis.ScanSeqs | | |
| --- | --- | --- |
| `public static final int` | `FDR_ID` | `5` |
| `public static final int` | `PVALUE_ID` | `3` |
| `public static final int` | `QVALUE_ID` | `4` |
| `public static final int` | `SCORE_ID` | `0` |
| `public static final int` | `SUMC_ID` | `2` |
| `public static final int` | `SUMT_ID` | `1` |

| edu.utah.seq.data.EnrichedRegion | | |
| --- | --- | --- |
| `public static final long` | `serialVersionUID` | `2L` |

| edu.utah.seq.data.Info | | |
| --- | --- | --- |
| `public static final long` | `serialVersionUID` | `1L` |

| edu.utah.seq.data.SmoothingWindow | | |
| --- | --- | --- |
| `public static final long` | `serialVersionUID` | `1L` |

| edu.utah.seq.data.SmoothingWindowInfo | | |
| --- | --- | --- |
| `public static final long` | `serialVersionUID` | `2L` |

| edu.utah.seq.misc.MakeUSeq | | |
| --- | --- | --- |
| `public static final java.lang.String` | `menuHeader` | `"<html><head><title>USeq Command Line Menus</title><style type=\"text/css\">#rt{text-align:right; color: #000000; font-weight: bold}#grBk {background-color: #CC9966;}TD {font-family: Verdana, Arial, Helvetica, sans-serif; font-size:12;}H1 {color: #996633; font:arial; font-size:16;}H2 {color: #996633; font:arial; font-size:12;}BODY {color:black; background-color:white; font-family: Verdana, Arial, Helvetica, sans-serif; font-size:12;}A:link {text-decoration: none; color: #000000; font-weight: bold} A:visited {text-decoration: none; color: #000000; font-weight: bold} A:hover {text-decoration: none; color: #FFCC66; font-weight: bold} A:active {text-decoration: none; color: #000000; font-weight: bold} </style></head><body><H1>Command Line Menus</H1> <p>"` |

| edu.utah.seq.parsers.BarParser | | |
| --- | --- | --- |
| `public static final java.lang.String` | `BP_3_PRIME_SHIFT` | `"bp3PrimeShift"` |
| `public static final java.lang.String` | `DESCRIPTION_TAG` | `"description"` |
| `public static final java.lang.String` | `GRAPH_MAX_Y_TAG` | `"initialMaxY"` |
| `public static final java.lang.String` | `GRAPH_MIN_Y_TAG` | `"initialMinY"` |
| `public static final java.lang.String` | `GRAPH_TYPE_BAR` | `"Bar"` |
| `public static final java.lang.String` | `GRAPH_TYPE_COLOR_TAG` | `"initialColor"` |
| `public static final java.lang.String` | `GRAPH_TYPE_DOT` | `"Dot"` |
| `public static final java.lang.String` | `GRAPH_TYPE_HEATMAP` | `"Heat Map"` |
| `public static final java.lang.String` | `GRAPH_TYPE_LINE` | `"Line"` |
| `public static final java.lang.String` | `GRAPH_TYPE_MINMAXAVE` | `"Min/Max/Ave"` |
| `public static final java.lang.String` | `GRAPH_TYPE_STAIRSTEP` | `"Stairstep"` |
| `public static final java.lang.String` | `GRAPH_TYPE_TAG` | `"initialGraphStyle"` |
| `public static final java.lang.String` | `READ_LENGTH_TAG` | `"readLength"` |
| `public static final java.lang.String` | `SOURCE_TAG` | `"source"` |
| `public static final java.lang.String` | `STRAND_TAG` | `"strand"` |
| `public static final java.lang.String` | `UNIT_TAG` | `"unit"` |
| `public static final java.lang.String` | `WINDOW_SIZE` | `"windowSize"` |

trans.bkgrnd.\*

| trans.bkgrnd.MapControlIntensities | | |
| --- | --- | --- |
| `public static final float` | `TARGET_MEDIAN` | `50.0f` |

trans.tpmap.\*

| trans.tpmap.IntensityFeature | | |
| --- | --- | --- |
| `public static final long` | `serialVersionUID` | `1L` |

| trans.tpmap.MapFeature | | |
| --- | --- | --- |
| `public static final long` | `serialVersionUID` | `3L` |

| trans.tpmap.MummerMapper | | |
| --- | --- | --- |
| `public static final java.lang.String` | `controlChromosomeName` | `"chrCtrls"` |

util.amazon.\*

| util.amazon.S3CodeSamples | | |
| --- | --- | --- |
| `public static final java.lang.String` | `AWS_ACCESS_KEY_PROPERTY_NAME` | `"0A4PT2HMEBJF9EDSBX82"` |
| `public static final java.lang.String` | `AWS_SECRET_KEY_PROPERTY_NAME` | `"P4cb3zqbqG0p3xBDaGuhjcPAPIokAkRLrcTeXB3k"` |
| `public static final java.lang.String` | `SAMPLES_PROPERTIES_NAME` | `"samples.properties"` |

util.apps.\*

| util.apps.MakeBioTools | | |
| --- | --- | --- |
| `public static final java.lang.String` | `menuHeader` | `"<html><head><title>TiMAT2 Command Line Menus</title><style type=\"text/css\">#rt{text-align:right; color: #000000; font-weight: bold}#grBk {background-color: #CC9966;}TD {font-family: Verdana, Arial, Helvetica, sans-serif; font-size:12;}H1 {color: #996633; font:arial; font-size:16;}H2 {color: #996633; font:arial; font-size:12;}BODY {color:black; background-color:white; font-family: Verdana, Arial, Helvetica, sans-serif; font-size:12;}A:link {text-decoration: none; color: #000000; font-weight: bold} A:visited {text-decoration: none; color: #000000; font-weight: bold} A:hover {text-decoration: none; color: #FFCC66; font-weight: bold} A:active {text-decoration: none; color: #000000; font-weight: bold} </style></head><body><H1>Command Line Menus</H1> <p>"` |

util.bio.\*

| util.bio.seq.ConvertFasta2RealSeqBoolean | | |
| --- | --- | --- |
| `public static final java.lang.String` | `NAME_NUM_REAL_BASES_FILE` | `"numberRealBases.txt"` |

| util.bio.wrappers.Primer3Pick | | |
| --- | --- | --- |
| `public static final java.lang.String` | `header` | `"PRIMER_LEFT_SEQUENCE\tPRIMER_RIGHT_SEQUENCE\tPRIMER_LEFT_TM\tPRIMER_RIGHT_TM\tPRIMER_PRODUCT_SIZE\tSequence\t"` |

---


|  |  |  |
| --- | --- | --- |
| **Overview** \| Package \| Class \| Use \| **Tree** \| **Deprecated** \| **Index** \| **Help** | |  |
| PREV   NEXT | **FRAMES**    **NO FRAMES**     **All Classes** |  |


---
